# Physician knowledge, attitudes, and perceptions of respiratory syncytial virus in older adults: A cross-sectional survey in Germany and Italy

**DOI:** 10.1371/journal.pone.0330763

**Published:** 2025-08-28

**Authors:** Anna Puggina, Alen Marijam, Olivier Cailloux, Marta Vicentini, Elisa Turriani, Frederik Verelst, Theo Last, Christina Rieger, Paolo Bonanni, Chiara de Waure, Pavo Marijic, Tommi Tervonen

**Affiliations:** 1 GSK, Verona, Italy; 2 GSK, Wavre, Belgium; 3 Kielo Research, Zug, Switzerland; 4 LAMSADE, CNRS, Université Paris-Dauphine, Université PSL, Paris, France; 5 Hematology Onkology Germering, Germering, Germany and University of Munich, Munich, Germany; 6 Dipartimento di Scienze della Salute, Università degli Studi di Firenze, Florence, Italy; 7 Dipartimento di Medicina e Chirurgia, Università degli Studi di Perugia, Perugia, Italy; 8 GSK, Munich, Germany; Carol Davila University of Medicine and Pharmacy, ROMANIA

## Abstract

Respiratory syncytial virus (RSV) causes substantial morbidity and mortality among older adults and individuals with chronic diseases, putting pressure on healthcare systems. RSV vaccines became available in 2023, however, physicians’ knowledge and attitudes regarding RSV and RSV vaccines may hinder uptake. We aimed to understand physicians’ knowledge, attitudes, and perceptions of respiratory infections, including RSV, and vaccination. A pre-tested cross-sectional survey was fielded with physicians who commonly administer or recommend respiratory vaccines in Germany and Italy. Regression models assessed physicians’ characteristics associated with knowledge and perceptions of RSV. Overall, 307 physicians (Germany: n = 152; Italy: n = 155) completed the survey. Physicians had good mean RSV disease knowledge scores (Germany: 3.8/5; Italy: 3.3/5). Most (Germany: 68%; Italy: 72%) indicated wanting further information on RSV, versus <35% for other respiratory diseases. Most considered RSV an important burden, especially for adults aged ≥50 years with immunocompromising conditions (Germany: >96%; Italy: >92%) and ≥60 years with chronic conditions (Germany: >95%; Italy: >87%). Physicians seeing >100 versus ≤100 patients weekly, and general practitioners versus other specialists, perceived RSV burden as more important. Perceived barriers to RSV vaccination included lack of national recommendation and reimbursement (Germany: 92%; Italy: 89%), and patients not being informed on getting vaccinated (Germany: 71%; Italy: 59%). In conclusion, physicians generally considered RSV an important pathogen for older adults and those with chronic conditions at risk of severe infection. Most wanted more information on RSV, thus medical education is important to address knowledge gaps and enable physicians to guide patients in making informed vaccination decisions.

## Introduction

Respiratory syncytial virus (RSV) infection causes substantial morbidity and mortality, and is associated with significant healthcare resource use [1–3]. RSV infections are common in adults, and the risk of developing severe complications is greater in older adults and those with comorbidities, likely due to a greater degree of immunocompromising conditions or changes in lung or heart physiology in these populations [4].

Varying estimates of RSV incidence rates have been reported globally, ranging from 6.7–‍16.2/1,000 persons annually [1–3]. A high disease burden has been reported in the European Union, with >270,000 RSV-associated hospitalizations estimated among adults aged ≥60 years in 2019, including 34,421 hospitalizations in Germany and 26,262 in Italy [3]. The burden of RSV is expected to increase due to the aging population and a growing proportion of older adults suffering from chronic conditions [5]. However, knowledge of the burden of RSV disease in older adults is low among physicians, given the underdiagnosis of RSV due to the lack of routine testing. Even when RSV is tested for or surveilled, variations in testing methods and lack of appropriate case definitions contribute to the underestimation of the true disease burden [6–8].

Treatment of RSV infection is primarily supportive, and there currently are no antiviral treatment options for RSV among adults [1,9]. For decades, RSV has also been an elusive target for vaccination, with several vaccine trials proving unfruitful [10]. The recent success of three vaccines against RSV in older adults therefore represents a significant milestone in RSV prevention [11–13]. In 2023, the adjuvanted prefusion conformation stabilized RSV F protein subunit vaccine (RSVPreF3) and bivalent RSV prefusion F subunit vaccine (RSVpreF) were licensed for use in older adults in Europe and the United States (US) [13–17]. In May 2024, an mRNA-based vaccine targeting the stabilized RSV prefusion F glycoprotein (mRNA-1345; mRESVIA) was approved for older adults by the US Food and Drug Administration, and received a positive opinion from the European Medicines Agency Committee for Medicinal Products in June 2024 [13,18,19]. However, physicians’ knowledge and attitudes regarding RSV may present a barrier to vaccine uptake, especially given the lack of knowledge surrounding RSV disease burden.

Physicians have consistently been recognized as a key source of information on vaccines and an important influence on vaccination decisions in risk groups [20–22]. However, existing literature indicate that current physician knowledge regarding respiratory infections and vaccine recommendations is suboptimal [23–25]. For example, it has been observed that Italian physicians have unsatisfactory understanding of the occurrence and potential consequences of RSV in the elderly [23]. In addition, a lack of information on vaccines is frequently reported among Italian physicians as a reason not to recommend respiratory vaccines [25]. Improving physician awareness and knowledge of the RSV disease burden and vaccination may therefore reduce barriers to vaccination and improve vaccine uptake in at-risk populations.

**Fig 1:**
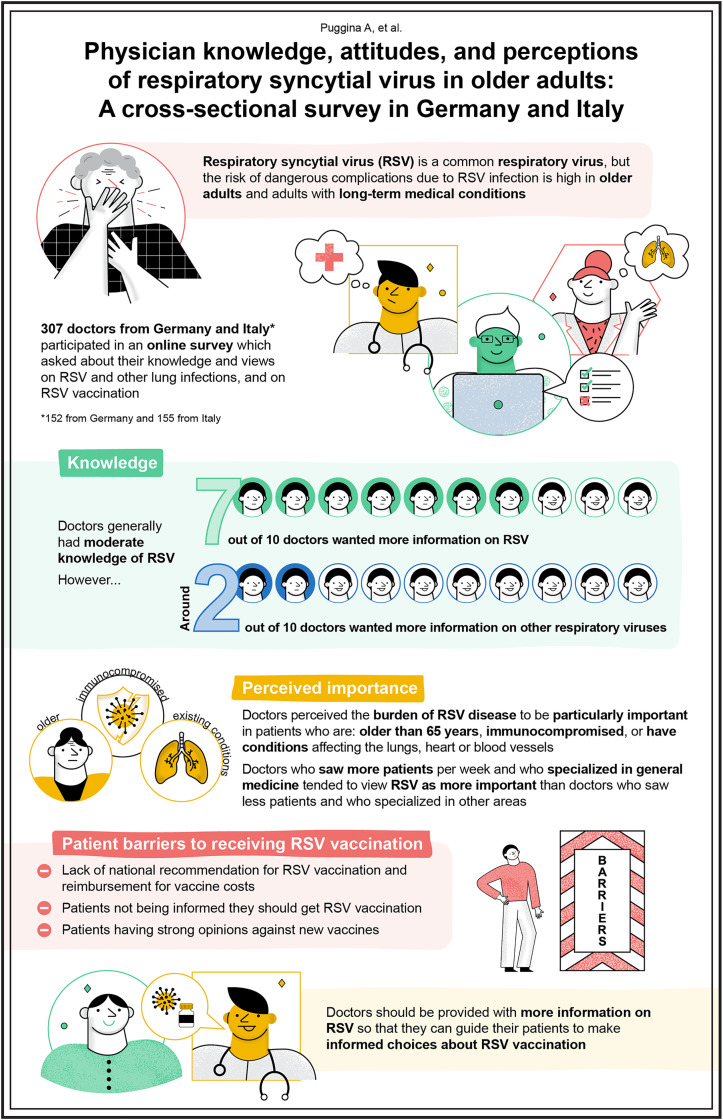
Graphical plain language summary. RSV: respiratory syncytial virus.

This study aimed to characterize the knowledge, attitudes, and perceptions of RSV and other respiratory infections (pertussis, pneumococcus, and influenza), and identify potential barriers to RSV vaccination among physicians in Germany and Italy. These countries were selected to represent different healthcare delivery models: in Germany, vaccination is administered in the outpatient setting by both specialists and general practitioners (GPs), who are mostly based in the private sector, whereas in Italy, vaccination is administered by GPs and public health specialists, who are all based in the public sector, while other specialists can only recommend vaccination to their patients. We further aimed to evaluate key physician characteristics associated with their perceptions of RSV burden and barriers to RSV vaccination. A graphical plain language summary of the study results is provided in **Fig 1**.

## Materials and methods

### Study design and survey

A cross-sectional survey-based study was performed from 11^th^ April to 29^th^ May 2023. A targeted literature review was first conducted to understand the knowledge, attitudes, and perceptions on non-pandemic respiratory diseases and vaccines against them in Germany and Italy, and commonly used measures to survey this. The results informed the development of the survey, which was specifically designed for the purposes of this study. Prior to administration, the survey was pre-tested in cognitive pilot interviews (n = 4 German physicians and n = 4 Italian physicians). The survey was translated into German and Italian via forward translation, and local language versions were used for data collection. The survey was disseminated via web-based survey software; a web-based approach was selected in alignment with the identified literature [25–27]. The English version of the survey is provided in S1 Appendix in S1 File.

Briefly, the survey included screening questions to assess participants’ study eligibility, an informed consent form, a sociodemographic questionnaire, and study-specific questions. Questions to test physicians’ knowledge of respiratory vaccinations and RSV infection and disease were presented as true/false statements. Answers to these questions were used to calculate knowledge scores on a scale of 0–4 for respiratory vaccine recommendations and 0–5 for RSV infection and disease, where 0 indicated no knowledge and the highest score indicated perfect knowledge. Likert-scale questions were used to capture physicians’ attitudes and perceptions on respiratory illness diagnosis and management, the importance of RSV burden in various patient subpopulations, and barriers that might prevent patients from receiving RSV vaccination. As RSV vaccinations were not approved by the European Medicines Agency at the time of the survey, physicians were asked to estimate the potential extent to which given factors would act as a potential barrier to RSV vaccination.

### Participant recruitment

Potentially eligible participants were identified via physician panels and were sent a pre-defined recruitment message containing a link to the final survey. Interested participants then self-completed the screener and the survey. Only data from fully completed surveys were included in this analysis.

This study recruited physicians who were GPs and physicians in other specializations, including pulmonology (Germany or Italy), diabetology (Germany), cardiology (Germany), hygiene and public health (Italy), and infectious disease (Italy). Physicians in these specializations were selected as they were considered those who may commonly administer or recommend respiratory vaccines. Physicians specializing in pulmonology, diabetology, and cardiology may also provide care for patients with chronic conditions who are particularly vulnerable to RSV illness.

Soft quotas were targeted for geographical regions and clinical specialties, informed by country population split across regions (S2 Appendix in S1 File). Participants were remunerated after fully completing the survey.

### Statistical calculations and data analysis

To ensure sufficient statistical power, the study aimed to include 300 physicians. The width of the 95% confidence interval (CI) for averages of continuous variables for physicians within a given country (n = 150 per country) is 0.32 times the standard deviation (SD) of the variable. The smallest significant analysis unit was the comparison of GPs and specialists within a country (n = 75 per comparison group). Assuming a medium effect size of half the geometric mean of each group’s SD, a Welch t-test has 86% power to detect between-group differences at a 0.05 significance level.

Descriptive analyses were conducted for the overall physician sample and for subgroups (Germany versus Italy and GPs versus specialists). Differences in categorical attributes were assessed using Pearson’s chi-squared test or Fisher’s exact test. Significance of differences in continuous variables were assessed using Wilcoxon rank sum or Welch t-test. All statistical tests were two-sided. All analyses were conducted using R version 4.2. P values of 0.05 or less were considered to indicate statistical significance. P values were not adjusted for multiple testing.

Multivariable linear regression models were developed to assess the association between physician characteristics and their knowledge of respiratory vaccine recommendations and RSV disease. In these models, the target variable was knowledge score, and the pre-specified explanatory variables included country, sex, specializations, main place of work, years of experience, and patients seen per week. Multivariable ordinal regression models were also developed to analyze the association of key physician characteristics and their perceptions of RSV burden, and potential barriers to RSV vaccination. Explanatory variables were the same as in the linear regression models, with the addition of knowledge of respiratory vaccine recommendations (score 0–4) and knowledge of RSV (score 0–5).

All survey results are reported descriptively and separately for Germany and Italy. Regression model results are reported for the overall population, controlling for the differences by country.

### Ethics statement

All participants provided written informed consent via an informed consent form at the beginning of their survey. This study was granted exemption from ethical oversight by Salus Institutional Review Board (study identifier: 23039−01) and complied with all applicable laws regarding participant privacy.

## Results

### Participant disposition and characteristics

The physician sample disposition is presented in S1 Fig in S1 File. A total of 15,000 members of physician panels were invited to complete the survey. Of these, 831 opened the survey, 321 were eligible and consented, and 307 physicians completed the survey and were included in the analysis (152 physicians from Germany and 155 physicians from Italy).

The sociodemographic characteristics of respondents are listed in **Table 1**. Overall, the mean age was 55.1 years old (SD: 10.4), most respondents were male (72%), had more than 20 years’ experience (66%), and saw > 100 patients per week (55%). The most frequently reported specialties were general practice (46%), followed by pulmonology (29%) and cardiology (15%).

**Table 1:** Sociodemographic characteristics of physician respondents.

Characteristic	Overall (N = 307)	Germany (n = 152)	Italy (n = 155)	p value^†^
Age, years				0.631
Mean (SD)	55.1 (10.4)	56.4 (7.2)	53.9 (12.7)	
Sex, n (%)				0.815
Male	220 (71.7)	108 (71.1)	112 (72.3)	
Female	87 (28.3)	44 (28.9)	43 (27.7)	
Main place of work, n (%)				<0.001
Private practice	126 (41.0)	118 (77.6)	8 (5.2)	
Public clinic	73 (23.8)	16 (10.5)	57 (36.8)	
Private hospital	19 (6.2)	4 (2.6)	15 (9.7)	
Public hospital	83 (27.0)	14 (9.2)	69 (44.5)	
Vaccination center	5 (1.6)	0 (0)	5 (3.2)	
Other	1 (0.3)	0 (0)	1 (0.6)	
Experience, n (%)				<0.001
0–5 years	8 (2.6)	1 (0.7)	7 (4.5)	
6–10 years	21 (6.8)	2 (1.3)	19 (12.3)	
11–20 years	74 (24.1)	46 (30.3)	28 (18.1)	
More than 20 years	204 (66.4)	103 (67.8)	101 (65.2)	
Patients per week, n (%)				0.008
0–50	20 (6.5)	4 (2.6)	16 (10.3)	
50–100	117 (38.1)	54 (35.5)	63 (40.6)	
More than 100	170 (55.4)	94 (61.8)	76 (49.0)	
Specialization,^‡^ n (%)				N/A
General practice	142 (46.3)	84 (55.3)	58 (37.4)	
Pulmonology	88 (28.7)	34 (22.4)	54 (34.8)	
Cardiology	47 (15.3)	35 (23.0)	12 (7.7)	
Diabetology	25 (8.1)	16 (10.5)	9 (5.8)	
Hygiene and public health	34 (11.1)	0 (0)	34 (21.9)	
Infectious disease	40 (13.0)	6 (3.9)	34 (21.9)	

† Italy versus Germany; ^‡^Respondents were able to report multiple specializations. Statistical analyses were performed with Wilcoxon rank sum test for continuous variables and Pearson’s chi-squared test or Fisher’s exact test for categorical variables. Statistical significance was taken at p < 0.05. N/A: not applicable; SD: standard deviation.

Cross-country differences were observed for the following physician characteristics: main place of work, duration of clinical experience, and number of patients seen per week. The majority of physicians from Germany worked in private practice, whereas most in Italy worked in public hospitals. In addition, there were more physicians with >11 years’ experience and who saw > 100 patients a week in Germany compared to Italy (**Table 1**).

### Knowledge of respiratory vaccination and RSV

**Fig 2:**
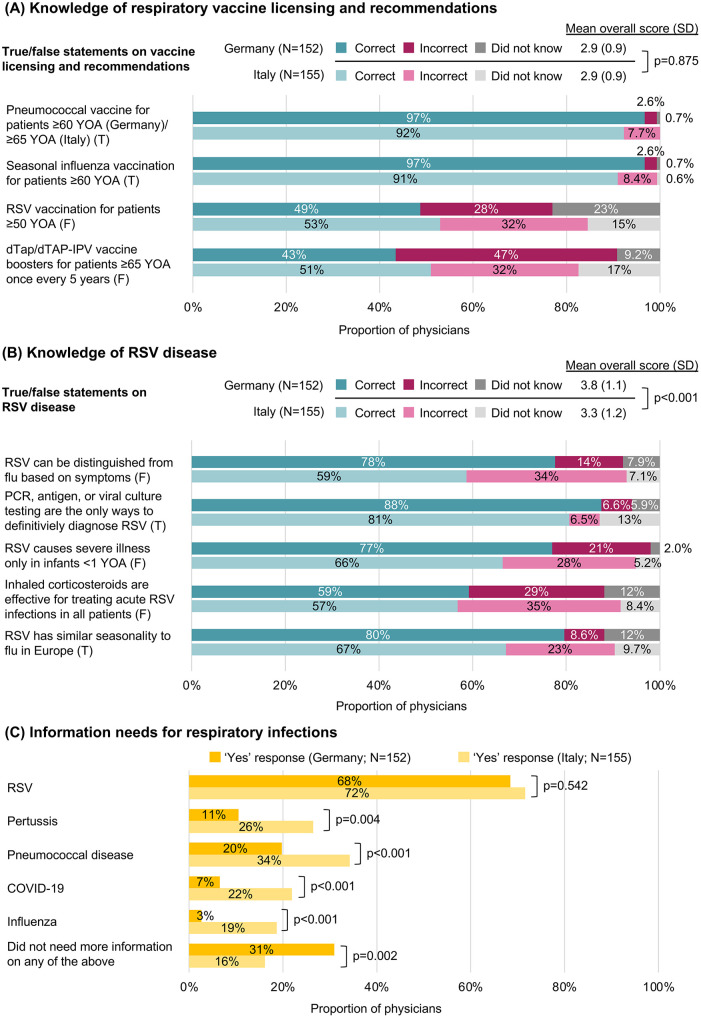
Physician knowledge and information needs. (A) Physician knowledge of respiratory vaccine recommendations based on responses to true/false statements. Knowledge scores range from 0–4, with 0 indicating no knowledge and 4 indicating perfect knowledge. Pneumococcal vaccines are licensed and recommended for adults ≥60 YOA in Germany and ≥65 YOA in Italy, and the seasonal influenza vaccine for adults ≥60 YOA in both countries. RSV vaccines were not licensed nor recommended at the time of the study. dTap is only recommended for children and adolescents for pertussis. For adults in Germany, a single Tdap booster is recommended for those ≥18 YOA, however there is no recommendation for those ≥65 YOA. In Italy, dTap boosters are recommended every 10 years in those ≥18 YOA. (B) Physician knowledge of RSV disease based on responses to true/false statements. Correct answers to knowledge statements are indicated in brackets next to the statement. Knowledge scores range from 0–5, with 0 indicating no knowledge and 5 indicating perfect knowledge. (C) Physician responses to whether information on a particular respiratory infection was needed. More than one option could be selected. Significant differences were assessed with Wilcoxon rank sum or Welch t-test; p-values were not adjusted for multiple testing. dTaP: diphtheria, tetanus, and pertussis; dTaP-IPV: diphtheria, tetanus, and pertussis combined with inactivated poliovirus; F: false; PCR: polymerase chain reaction; RSV: respiratory syncytial virus; SD: standard deviation; T: true; Tdap: tetanus, diphtheria, pertussis; YOA: years of age.

Mean (SD) knowledge scores of respiratory vaccine license statuses and clinical recommendations were 2.9 (0.9) for both physicians from Germany and from Italy. Most physicians were aware of the licensing and recommendations of pneumococcal vaccines (Germany: 97%; Italy: 92%) and seasonal influenza vaccines (Germany: 97%; Italy: 91%) in their respective countries (**Fig 2A**) [28,29]. Approximately half of physicians correctly identified the false statement that RSV vaccines were licensed and recommended for adults aged ≥50 years at the time of the study (Germany: 49%; Italy: 53%). A similar proportion correctly identified the false statement that diphtheria, tetanus, and pertussis (dTaP) or dTaP combined with inactivated poliovirus (dTaP-IPV) vaccines are licensed and recommended for adults aged ≥65 years every five years (Germany: 43%; Italy: 51%), as these vaccines are recommended in children and adolescents only.

Multivariable linear regression models assessing the effect of physician characteristics on knowledge of respiratory vaccination recommendations revealed that no particular specialization had a significant effect on such compared with other specializations, except for specialization in infectious diseases, which was associated with greater knowledge (S1 Table in S1 File). No differences in knowledge of respiratory vaccination recommendations were observed between physicians from Germany and Italy.

The mean (SD) knowledge score of RSV disease was significantly higher in physicians from Germany than physicians from Italy (3.8 [1.1] vs 3.3 [1.2]; p < 0.001). The majority of physicians from both countries provided correct answers to all of the knowledge statements regarding RSV disease. A numerically greater proportion of physicians from Germany versus Italy correctly asserted that: flu cannot be distinguished from RSV based on symptoms (Germany: 78%; Italy: 59%), that polymerase chain reaction (PCR), antigen, or viral culture testing are the only ways by which RSV can be diagnosed definitively (Germany: 88%; Italy: 81%), that RSV does not cause severe illness in infants only (Germany: 77%; Italy: 66%), that inhaled corticosteroids are not effective in treating acute respiratory infections caused by RSV in all patients (Germany: 59%; Italy: 57%), and that RSV has similar seasonality to flu in Europe (Germany: 80%; Italy: 67%) (**Fig 2B**). Multivariable linear regression results indicated that, compared with other specializations, specialization in cardiology and infectious disease was associated with greater knowledge of RSV disease, whereas specialization in hygiene and public health was associated with poorer knowledge (S2 Table in S1 File).

Most physicians indicated a need for information on one or more respiratory infections, with the highest need on RSV in 68% of physicians from Germany and 72% from Italy (**Fig 2C**). Significant cross-country differences were observed whereby fewer physicians from Germany versus Italy wanted further information on influenza (2.6% vs 19%; p < 0.001), COVID-19 (6.6% vs 22%; p < 0.001), pertussis (20% vs 34%; p = 0.004), and pneumococcal disease (11% vs 26%; p < 0.001). A minority of physicians (Germany: 31%; Italy: 16%; p = 0.002) reported that they did not need more information on any of these respiratory infections.

Responses on information needs by specialization among physicians who reported a single specialization are presented in S3 Table in S1 File. The highest information need was on RSV, reported by more than half of physicians in each specialization.

### Attitudes on vaccination and RSV

Overall, patient vaccination status was evaluated either at every visit or often by 80% of physicians for influenza, 75% for COVID-19, 72% for pneumococcal disease, and 44% for pertussis. More than half of physicians (54%) strongly or somewhat agreed that they rarely considered RSV as a potential cause of illness in patients presenting with flu-like symptoms. Less than half do not test for RSV due to the lack of available treatment (39%), costs of testing (27%), or long test duration (24%). When comparing influenza versus RSV infection outcomes in patients aged ≥60 years, under a third of physicians strongly or somewhat agreed that RSV causes less severe symptoms (22%), fewer hospitalizations (32%), fewer deaths (32%), and that having an RSV vaccine would not be as important as an influenza vaccine in these patients (24%).

### Perceptions of RSV importance

**Fig 3:**
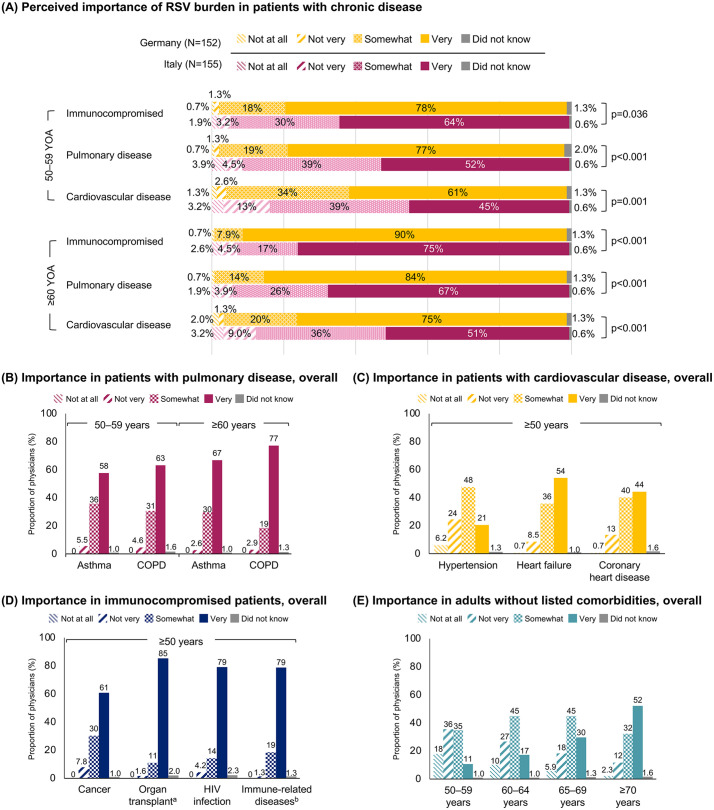
Physician perceived importance of RSV burden in different patient populations. (A) Perceived importance in patients with chronic disease is reported by country. (B–E) Perceived importance in patients with pulmonary disease, cardiovascular disease, and immunocompromising diseases, and adults without the listed comorbidities are reported for the overall population (Germany and Italy combined; see S4 Table in S1 File for results split by country). Significant differences were assessed with Wilcoxon rank sum or Welch t-test; p-values were not adjusted for multiple testing. ^a^Patients who have received an organ transplant and currently receiving immunosuppressant therapy. ^b^Patients treated with any immunosuppressant drug for immune-related disease such as rheumatoid arthritis or psoriasis. RSV: respiratory syncytial virus.

Physicians generally assessed the RSV burden to be important among patients who are immunocompromised, or with chronic pulmonary or cardiovascular disease, particularly among older patients aged ≥60 years (Fig 3 and S4 Table in S1 File). More than 66% of physicians from Germany and ≥45% from Italy perceived RSV to be a very important burden for patients aged ≥50 years with lung and immunocompromising conditions. A particularly high proportion considered RSV to be very important among patients aged ≥50 years with an HIV infection or who are on immunosuppressant therapy (>90% of physicians from Germany and >66% from Italy). While 41–58% of physicians across both countries perceived RSV to be very important for patients with heart failure and coronary heart disease, fewer physicians expressed the same sentiment for patients with hypertension (Germany: 22%; Italy: 19%).

Of note, a numerically higher proportion of physicians from Germany than Italy responded that RSV was very important for all patient groups included in the survey (S4 Table in S1 File). Several significant cross-country differences were noted, with more physicians in Germany vs Italy (p < 0.001 for all) responding that RSV was very important in patients with asthma (50–59 years: 70% vs 45%; ≥60 years: 78% vs 55%) or chronic obstructive pulmonary disease (COPD; 50–59 years: 77% vs 50%; ≥60 years: 86% vs 68%), and patients aged ≥50 years with HIV infection (91% vs 68%) or being treated with an immunosuppressant drug for an immune-related disease (92% vs 66%).

In contrast, only 11% of physicians from Germany and 10% from Italy considered RSV to be very important for adults aged 50–59 years without the listed comorbidities (S4 Table in S1 File). However, these proportions increased with the age of the patient, and 58% of physicians from Germany and 46% from Italy considered RSV to be very important for adults ≥70 years of age without the listed comorbidities.

**Fig 4:**
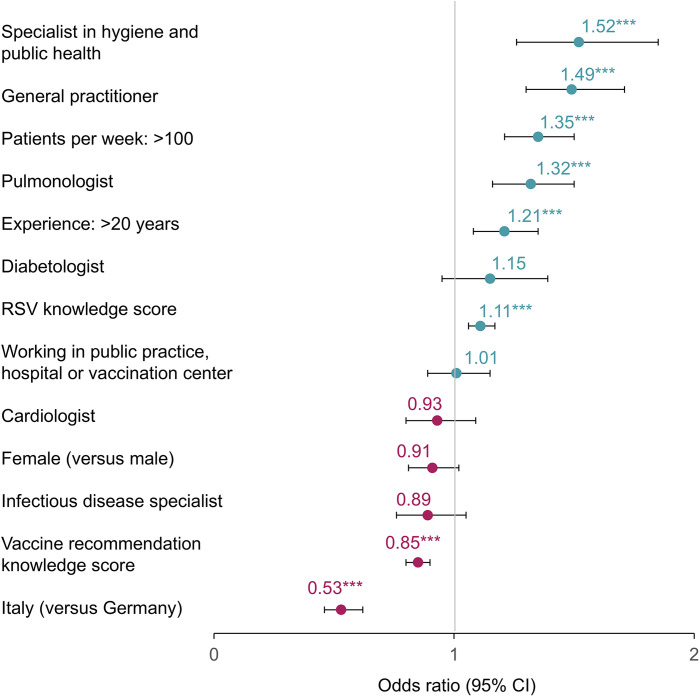
Effect of physicians’ characteristics on their perceived importance of RSV. Multivariable ordinal regression model. ***p < 0.001. CI: confidence interval; RSV: respiratory syncytial virus.

Corresponding multivariable ordinal regression modelling indicated that the perceived importance of RSV differed by physician characteristics (**Fig 4**). General practitioners (odds ratio: 1.49 [95% CI: 1.30–1.71]), specialists in hygiene and public health (1.52 [1.26–1.85]), and pulmonologists (1.32 [1.16–1.50]) tended to perceive RSV as important (p < 0.001 for all). Physicians seeing >100 patients per week versus ≤100 patients (1.35 [1.21–1.50]), with >20 years’ experience versus ≤20 years (1.21 [1.08–1.35]), and with greater knowledge of RSV disease (1.11 [1.06–1.17]) also tended to have higher perceived importance of RSV (p ≤ 0.001 for all). However, greater knowledge of vaccination recommendations was associated with lower perceived importance of RSV (0.85 [0.80–0.90]; p < 0.001). Physicians from Italy tended to perceive RSV as less important compared with physicians from Germany (0.53 [0.46–0.62]; p < 0.001).

### RSV vaccination barriers

**Fig 5:**
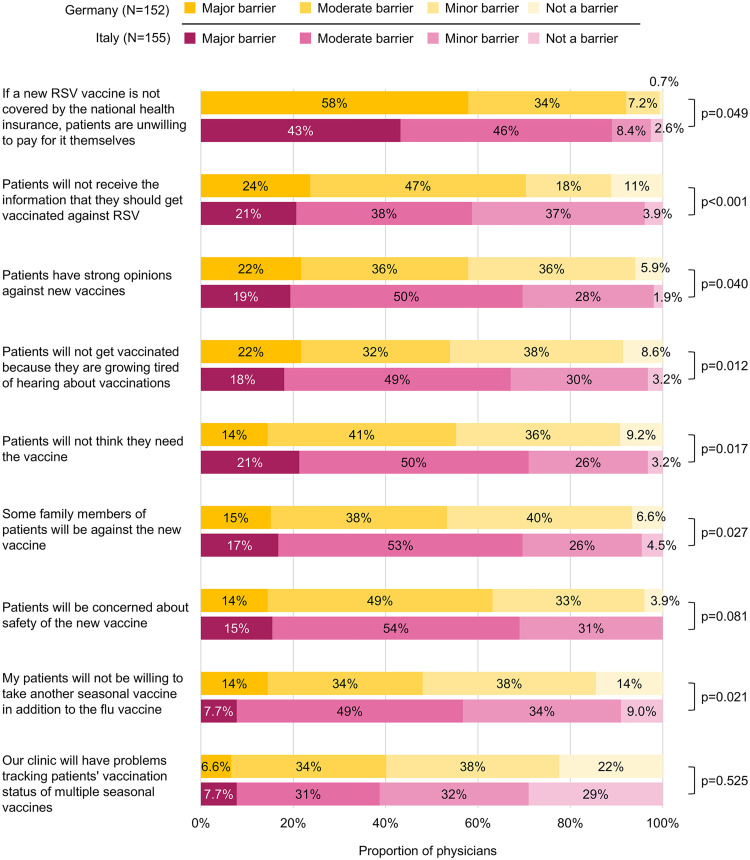
Physician perceptions of barriers against RSV vaccination. RSV: respiratory syncytial virus.

Although RSV vaccines were not licensed in Europe at the time of the survey, many physicians indicated that a lack of national recommendation and reimbursement would potentially be the main barrier for RSV vaccination. The majority of physicians believed patients would not be willing to pay for a new RSV vaccine if it was not nationally reimbursed (major or moderate barrier; Germany: 92%; Italy: 89%), with approximately half of the physicians identifying this as a major barrier against RSV vaccination (Germany: 58%; Italy: 43%; **Fig 5**). A minority of physicians considered their clinic’s ability to track patients’ multiple vaccination status as a major or moderate barrier to vaccination (Germany: 40%; Italy: 39%).

More physicians in Germany than Italy perceived patients not receiving the information that they should get vaccinated against RSV as a major or moderate barrier (70% vs 59%; p < 0.001). In contrast, slightly fewer physicians in Germany than Italy considered patient concerns over safety (63% vs 69%; p = 0.081), strong opinions against new vaccines in patients (58% vs 70%; p = 0.040) or their family members (53% vs 70%; p = 0.027), and vaccine fatigue (54% vs 67%; p = 0.012) as major or moderate barriers to RSV vaccination. Multivariable ordinal regression model results on the effects of physician characteristics on perceived barriers to RSV vaccination are summarized in S5 Table in S1 File.

## Discussion

In this study, we characterized knowledge, attitudes, and perception of respiratory infections, including RSV, among physicians in Germany and Italy. We further evaluated if specific physicians’ characteristics were associated with their knowledge of respiratory vaccinations and RSV disease, and perceptions of RSV burden and barriers of RSV vaccination.

Although RSV vaccines were not licensed nor recommended in Germany and Italy at the time of the study, physicians generally had good knowledge of RSV disease, such as diagnosis methods and treatment options, as demonstrated by a mean knowledge score of 3.8 in Germany and 3.3 in Italy (scale 0–5). Nevertheless, over two thirds of physicians from Germany and Italy reported wanting further information on RSV, while under a third of physicians reported wanting further information on pertussis, pneumococcal disease, or influenza. These findings may be due to the historical underassessment of RSV in older adults and, until recently, lack of preventive measures against RSV [9]. An earlier survey performed in a limited sample of physicians from the US similarly indicated large knowledge gaps on RSV in adults, particularly on the symptoms and risk factors [30]. Together, these results suggest a need for increased medical education to provide physicians with more information on RSV in adults.

In this study, while infectious disease specialists tended to have greater knowledge of respiratory vaccine recommendations compared with other specialists, no significant differences were observed for other specializations. However, the small sample size may have limited the precision of our regression models, and it could be that significant effects of specialization on physicians’ knowledge may be observed with a larger sample size. Furthermore, given that approval of RSV vaccines was first granted in Europe after the surveys were completed, it would be of interest to investigate physician knowledge of RSV vaccine recommendations post-approval [16,17].

Physicians generally considered RSV to be a somewhat or very important pathogen in patients aged ≥50 years with pulmonary disease, cardiovascular disease, who are immunocompromised, or in adults aged ≥60 years without these comorbidities. In particular, a majority of physicians considered RSV to impose a ‘very important’ burden on patients with immunocompromising conditions, such as HIV infection and being on immunosuppressant therapy, and pulmonary conditions, such as asthma and COPD. These results are consistent with global literature, in which older adults and those with comorbid conditions (e.g., cardiopulmonary or immunocompromising diseases) were found to be at higher risk of medically attended RSV infection [9,31]. Furthermore, a primary practitioner perspectives survey in the US has reported similar results [24], suggesting that physicians are generally aware of populations vulnerable to RSV infection.

Key factors affecting the perceived importance of RSV were physicians’ specialization, number of patients seen per week (≤100 versus >100), and years of experience (≤20 versus >20 years); physicians working as GPs, physicians specializing in hygiene and public health, pulmonologists, physicians who saw more patients per week, and more experienced physicians perceived RSV as more important. Although physicians from Italy reported lower perceived importance of RSV than physicians in Germany, this comparison should be interpreted with caution as cross-country differences in physicians’ characteristics, including main place of work, years of experience, and specialization, were not adjusted for but may have driven this difference. Greater physicians’ knowledge of RSV was also associated with higher perceived importance. In contrast, greater knowledge of vaccine recommendations was unexpectedly associated with lower perceived importance of RSV, which might indicate that physicians with lower knowledge of respiratory vaccine recommendations are generally more concerned with respiratory infections. Alternatively, the non-existence of RSV vaccine recommendations at the time of the survey may have led to lower perceived importance of RSV among physicians who were more knowledgeable of vaccine recommendations, warranting further investigation.

Most physicians identified the main barrier against RSV vaccination of older adults as a lack of national recommendation and reimbursement, consistent with a US survey of primary practitioner perspectives on RSV [24], despite cross-country (Germany, Italy, US) differences in healthcare systems and financing mechanisms. This suggests that providing recommendation and reimbursement for patients will be key to increase vaccine accessibility and decrease vaccine-preventable morbidity and mortality from RSV. Other barriers identified in this study, including lack of recommendation or information from physicians, concerns over vaccine safety, and perceived lack of need for vaccine have also been stated as barriers to adult influenza and pneumococcal vaccination [21,24,32,33].

Given the lack of viable treatment options for RSV in adults, high vaccine uptake and coverage is especially important to tackle the burden of RSV in this population [9]. Taken together with results from other studies, our findings call for a series of holistic interventions to be planned and implemented in order to facilitate RSV vaccination in older adult populations. The high information needs among physicians should be addressed with increased medical education efforts, ensuring physicians are well-informed on the latest information on RSV disease and vaccine recommendations. These efforts can further be supported by official recommendations made by scientific societies, such as a recent recommendation on RSV vaccination in adult risk groups made by four leading Italian societies [34], and National Immunization Technical Advisory Groups. By being better informed, physicians can better advise their patients on making informed vaccination decisions. Further, providing national recommendation and reimbursement for RSV vaccination may help increase accessibility, particularly for disadvantaged groups who are often at highest risk of severe RSV illness.

### Strengths and limitations

A strength of this study was that quotas were considered when recruiting participants, to enable measuring the effect of physicians’ specialization on their attitudes. We were therefore able to characterize how physicians’ knowledge and perceptions of RSV were affected by their demographics, such as specialization and clinical experience. The specialization quotas were set to reflect the type of physicians who typically administer or recommend vaccination in each country, which likely contributed to the differences seen in physician specialization and main place of work observed: > 75% of respondents from Germany mainly worked in private practice while >80% from Italy mainly worked in the public sector. Nevertheless, these differences limited cross-country comparisons of survey results.

A key limitation was that the study sample may have been subject to selection bias given the low response rates. Some imbalances in respondent characteristics were observed; for example, the majority were male and had >20 years of experience. These imbalances suggest that the study results may not be generalizable to physicians without these characteristics as they were underrepresented. Selection bias may also have occurred as participants were recruited from panels of physicians who have pre-opted to be included in online survey research. Nevertheless, quotas were applied to obtain populations that were representative in regional distribution and specialization. Other limitations inherent to the study design included the exclusion of potential participants without internet access and that results were self-reported. As the survey was completed by participants at their chosen time and place, they may have looked up correct answers to the knowledge questions. However, given participants were not rewarded for correct answers, they had no incentive to do so. Regarding data analysis, the regression analyses were performed with pooled data from Germany and Italy. Although country-specific differences were controlled for in the models, the explanatory variables may nevertheless have differential impacts on the knowledge and attitudes of physicians from the two countries.

## Conclusion

In conclusion, physicians in Germany and Italy generally considered RSV an important respiratory pathogen for the elderly and adults with immunocompromising conditions and comorbidities, who are at high risk of severe RSV. However, there were important knowledge gaps on RSV vaccination, and most physicians wanted more information on RSV and RSV vaccination. With the recent emergence of efficacious vaccines against RSV, it is therefore important to invest in education efforts so that physicians can provide adequate up-to-date information for patients to make informed vaccination decisions. Ensuring vaccine accessibility is also crucial to encourage vaccine uptake. These results may support decision makers on implementing national recommendations and reimbursements of RSV vaccines to decrease vaccine-preventable morbidity and mortality from RSV.

## Supporting information

S1 File Supplementary Materials. S1 Appendix. Sample physician survey in English. Local language versions (German or Italian) were used for data collection. S2 Appendix. Physician quotas targeted for the main survey phase. S1 Fig. Physician sample disposition. S1 Table. Effect of physicians’ characteristics on their knowledge of respiratory vaccination recommendations. S2 Table. Effect of physicians’ characteristics on their knowledge of RSV disease. S3 Table. Physician information needs of respiratory infections, by reported specialization. S4 Table. Physician perceived importance of RSV burden in different patient populations and adults without the listed comorbidities, by country. S5 Table. Effect of physician characteristics on perceived barriers to RSV vaccination.
(ZIP)
