# Supplementary material for: Physician knowledge, attitudes, and perceptions of respiratory syncytial virus in older adults: A cross-sectional survey in Germany and Italy
Source: PLoS One. 2025 Aug 28;20(8):e0330763. doi: 10.1371/journal.pone.0330763 (PMC12393788; doi:10.1371/journal.pone.0330763)
Supplement: S1 File — S1 Appendix. Sample physician survey in English. Local language versions (German or Italian) were used for data collection. S2 Appendix. Physician quotas targeted for the main survey phase. S1 Fig. Physician sample disposition. S1 Table. Effect of physicians’ characteristics on their knowledge of respiratory vaccination recommendations. S2 Table. Effect of physicians’ characteristics on their knowledge of RSV disease. S3 Table. Physician information needs of respiratory infections, by reported specialization. S4 Table. Physician perceived importance of RSV burden in different patient populations and adults without the listed comorbidities, by country. S5 Table. Effect of physician characteristics on perceived barriers to RSV vaccination. (ZIP) [file pone.0330763.s001.zip › Supporting_Information/S1_Appendix.docx]

Supplementary Appendix 1. Full physician survey (English version). Local language versions (German or Italian) were used for data collection.

# Screener

Do you work as a qualified doctor? *[implement as radio buttons.]*

- Yes
- No *[Exclude]*

Do you have training **and** practice any of the following (choose all that apply)?

- Cardiology
- Pulmonology
- Diabetology
- Infectious disease specialist
- Specialist in hygiene and public health *[Show for Italy only]*
- I work as a general practitioner
- None of the above *[Mutually exclusive with others; Exclude]*

*[Next page]*

In which region do you primarily work as a physician? *[Show list corresponding to country as radio buttons]*

| ***[Germany]*** | ***[Italy]*** |
| --- | --- |
| Baden-Württemberg | Abruzzo |
| Bayern | Basilicata |
| Berlin | Calabria |
| Brandenburg | Campania |
| Bremen | Emilia-Romagna |
| Hamburg | Friuli-Venezia Giulia |
| Hessen | Lazio |
| Mecklenburg-Vorpommern | Liguria |
| Niedersachsen | Lombardia |
| Nordrhein-Westfalen | Marche |
| Rheinland-Pfalz | Molise |
| Saarland | Piemonte |
| Sachsen | Puglia |
| Sachsen-Anhalt | Sardegna |
| Schleswig-Holstein | Sicilia |
| Thüringen | Toscana |
| I do not work in Germany [*exclude*] | Trentino Alto Adige/Südtirol |
|  | Umbria |
|  | Val D’Aosta |
|  | Veneto |
|  | I do not work in Italy *[exclude]* |

*Check specialization and geographical quotas, and exclude if over quota.*

# Survey

## Introduction

This survey consists of a few different sections.

We will ask questions to understand your knowledge, opinion, and experience of respiratory infections and vaccines to prevent them.

## Knowledge of respiratory vaccinations

The following statements are about respiratory vaccination. Please answer which you think are true or false statements.

*[Randomize order of questions for each participant – implement as radio buttons]*

|  | True | False | Don’t know |
| --- | --- | --- | --- |
| Pneumococcal vaccine is licensed and recommended for all patients >= 60 years old *[Show for Germany]* | * |  |  |
| Pneumococcal vaccine is licensed and recommended for all patients >= 65 years old *[Show for Italy]* | * |  |  |
| dTap/dTAP-IPV vaccine boosters against diphtheria, tetanus, and pertussis are licensed and recommended for patients >= 65 years old once every 5 years |  | * |  |
| RSV (respiratory syncytial virus) vaccine is licensed and recommended for all patients >= 50 years old |  | * |  |
| Seasonal influenza vaccination is licensed and recommended for all patients >= 60 years old | * |  |  |

## Knowledge of RSV

The following statements are about RSV (respiratory syncytial virus). Please answer which you think are true or false statements.

*[Randomize order of questions for each participant – implement as radio buttons]*

|  | True | False | Don’t know |
| --- | --- | --- | --- |
| Flu can be distinguished from RSV based on symptoms | * |  |  |
| Only way to diagnose RSV definitively is by PCR, antigen, or viral culture testing | * |  |  |
| RSV causes severe illness only in infants up to 1-years of age |  | * |  |
| Inhaled corticosteroids are effective for treating for acute respiratory infections caused by RSV in all patients |  | * |  |
| RSV has similar seasonality to flu in Europe | * |  |  |

## Perception of RSV burden in patients with chronic disease

How important is the RSV (respiratory syncytial virus) pathogen in different groups of patients with chronic conditions?

|  | Not at all important | Not very important | Somewhat important | Very important | Don’t know |
| --- | --- | --- | --- | --- | --- |
| Adults **50-59** years that are **immunocompromised** | □ | □ | □ | □ | □ |
| Adults **50-59** years with **pulmonary disease** | □ | □ | □ | □ | □ |
| Adults **50-59** years with **cardiovascular disease** | □ | □ | □ | □ | □ |
| Adults ≥ **60** years that are **immunocompromised** | □ | □ | □ | □ | □ |
| Adults ≥ **60** years with **pulmonary disease** | □ | □ | □ | □ | □ |
| Adults ≥ **60** years with **cardiovascular disease** | □ | □ | □ | □ | □ |

## Perception of RSV burden in patients with lung disease

How important is the RSV (respiratory syncytial virus) pathogen in different groups of patients with asthma or COPD?

|  | Not at all important | Not very important | Somewhat important | Very important | Don’t know |
| --- | --- | --- | --- | --- | --- |
| Adults **50-59** years with **asthma** | □ | □ | □ | □ | □ |
| Adults **50-59** years with **COPD** | □ | □ | □ | □ | □ |
| Adults ≥ **60** years with **asthma** | □ | □ | □ | □ | □ |
| Adults ≥ **60** years with **COPD** | □ | □ | □ | □ | □ |

## Perception of RSV burden in patients with cardiovascular disease

How important is the RSV (respiratory syncytial virus) pathogen in different groups of patients with cardiovascular disease?

|  | Not at all important | Not very important | Somewhat important | Very important | Don’t know |
| --- | --- | --- | --- | --- | --- |
| Adults 50 year or older with **hypertension** | □ | □ | □ | □ | □ |
| Adults 50 year or older with **heart failure** | □ | □ | □ | □ | □ |
| Adults 50 year or older with **coronary heart disease (CAD)** | □ | □ | □ | □ | □ |

## Perception of RSV burden in immunocompromised patients

How important is the RSV (respiratory syncytial virus) pathogen in immunocompromised patients?

|  | Not at all important | Not very important | Somewhat important | Very important | Don’t know |
| --- | --- | --- | --- | --- | --- |
| ≥ 50 years with **cancer** | □ | □ | □ | □ | □ |
| ≥ 50 years who have received an **organ transplant and currently receiving immunosuppressant therapy** | □ | □ | □ | □ | □ |
| ≥ 50 years with an **HIV infection** | □ | □ | □ | □ | □ |
| ≥ 50 years **treated with any immunosuppressant drug for immune related disease** (such as rheumatoid arthritis or psoriasis) | □ | □ | □ | □ | □ |

## Perception of RSV burden in healthy adults

How important is the RSV (respiratory syncytial virus) pathogen in healthy adults?

|  | Not at all important | Not very important | Somewhat important | Very important | Don’t know |
| --- | --- | --- | --- | --- | --- |
| Healthy **50-59** year old adults | □ | □ | □ | □ | □ |
| Healthy **60-64** year old adults | □ | □ | □ | □ | □ |
| Healthy **65-69** year old adults | □ | □ | □ | □ | □ |
| Healthy **70+** year old adults | □ | □ | □ | □ | □ |

## Perception of RSV

Do you agree or disagree with the following statements?

|  | **Strongly agree** | **Somewhat agree** | **Somewhat disagree** | **Strongly disagree** | **Don’t know** |
| --- | --- | --- | --- | --- | --- |
| When patient presents with flu-like symptoms, I rarely consider RSV as a potential cause of the illness | □ | □ | □ | □ | □ |
| I don’t test for RSV as there’s no available treatment | □ | □ | □ | □ | □ |
| I don’t test for RSV because of cost of the testing | □ | □ | □ | □ | □ |
| I don’t test for RSV as it takes too much time | □ | □ | □ | □ | □ |
| RSV causes less severe symptoms than flu in patients ≥ 60 year old | □ | □ | □ | □ | □ |
| RSV causes less hospitalizations than flu in patients ≥ 60 year old | □ | □ | □ | □ | □ |
| RSV causes less deaths than flu in patients ≥ 60 year old | □ | □ | □ | □ | □ |
| Having a vaccine for RSV would be not as important as having a vaccine for flu in patients ≥ 60 year old | □ | □ | □ | □ | □ |

## Barriers of RSV vaccination (1/2)

There are vaccines currently being developed for immunization of older adults against RSV.

How much of a barrier would you expect the following things to be for potential future vaccination of your **older adult** patients against RSV?

|  | **Major barrier** | **Moderate barrier** | **Minor barrier** | **Not at all a barrier** |
| --- | --- | --- | --- | --- |
| If a new RSV vaccine is not covered by the national health insurance, patients are unwilling to pay for it themselves | □ | □ | □ | □ |
| Patients will be concerned about safety of the new vaccine | □ | □ | □ | □ |
| Patients will not think they need the vaccine | □ | □ | □ | □ |
| Patients will not receive the information that they should get vaccinated against RSV | □ | □ | □ | □ |
| Patients have strong opinions against new vaccines | □ | □ | □ | □ |

## Barriers of RSV vaccination (2/2)

There are vaccines currently being developed for immunization of older adults against RSV.

How much of a barrier would you expect the following things to be for potential future vaccination of your **older adult** patients against RSV?

|  | **Major barrier** | **Moderate barrier** | **Minor barrier** | **Not at all a barrier** |
| --- | --- | --- | --- | --- |
| Some family members of patients will be against the new vaccine | □ | □ | □ | □ |
| Patients will not get vaccinated because they are growing tired of hearing about vaccinations | □ | □ | □ | □ |
| My patients will not be willing to take another seasonal vaccine in addition to the flu vaccine | □ | □ | □ | □ |
| Our clinic will have problems tracking patients’ vaccination status of multiple seasonal vaccines | □ | □ | □ | □ |

## Frequency of encountering respiratory infections

How often do you see older adult patients (≥ 60 years) with respiratory infections during the relevant season?

| **Respiratory infection** | **Daily** | **Weekly** | **Monthly** | **Less often than once every month** | **Don’t know** |
| --- | --- | --- | --- | --- | --- |
| Common cold | □ | □ | □ | □ | □ |
| Influenza | □ | □ | □ | □ | □ |
| COVID-19 | □ | □ | □ | □ | □ |
| Pneumococcal disease | □ | □ | □ | □ | □ |
| Pertussis | □ | □ | □ | □ | □ |
| RSV (respiratory syncytial virus) | □ | □ | □ | □ | □ |

## Vaccination status inspection frequency

How often do you check respiratory vaccination status of your older adult patients?

|  | **In every visit (always)** | **Often** | **Sometimes** | **Rarely** | **Never** |
| --- | --- | --- | --- | --- | --- |
| Influenza (during vaccination season) | □ | □ | □ | □ | □ |
| COVID-19 | □ | □ | □ | □ | □ |
| Pneumococcal disease | □ | □ | □ | □ | □ |
| Pertussis | □ | □ | □ | □ | □ |

## Respiratory infection information needs

Which of the following respiratory infections you feel you would need more information on? (check all that apply)

- Influenza
- COVID-19
- Pertussis
- Pneumococcal disease
- RSV (respiratory syncytial virus)
- I do not need more information on any of above *[Mutually exclusive with other options]*

## Sociodemographic form

We have just a few more questions for you.

What is your sex?

- Male
- Female

How old are you?

___ years *[Allow numbers only; prompt to re-state if <20 or >80]*

Where do you primarily work?

- Private practice
- Public clinic
- Private hospital
- Public hospital
- Vaccination centre
- Other: ___

How many years have you been practicing medicine?

- 0-5 years
- 6-10 years
- 11-20 years
- More than 20 years

How many patients do you see on average per week?

- 0-50
- 50-100
- More than 100

## End

Thank you for completing the survey!
