# Supplementary material for: Physician knowledge, attitudes, and perceptions of respiratory syncytial virus in older adults: A cross-sectional survey in Germany and Italy
Source: PLoS One. 2025 Aug 28;20(8):e0330763. doi: 10.1371/journal.pone.0330763 (PMC12393788; doi:10.1371/journal.pone.0330763)
Supplement: S1 File — S1 Appendix. Sample physician survey in English. Local language versions (German or Italian) were used for data collection. S2 Appendix. Physician quotas targeted for the main survey phase. S1 Fig. Physician sample disposition. S1 Table. Effect of physicians’ characteristics on their knowledge of respiratory vaccination recommendations. S2 Table. Effect of physicians’ characteristics on their knowledge of RSV disease. S3 Table. Physician information needs of respiratory infections, by reported specialization. S4 Table. Physician perceived importance of RSV burden in different patient populations and adults without the listed comorbidities, by country. S5 Table. Effect of physician characteristics on perceived barriers to RSV vaccination. (ZIP) [file pone.0330763.s001.zip › Supporting_Information/S2_Appendix.docx]

Supplementary Appendix 2. Physician quotas targeted for the main survey phase.

The following quotas were included to ensure representation from geographic regions and clinical specialties.

Geographical quota

The geographical quotas were designed to be proportionate to the population size of regions defined by the Nomenclature of Territorial Units for Statistics level 1 (NUTS-1)^1^ for Italy and a previous COVID-19 epidemiological analysis by the Robert Koch Institute for Germany.^2^

|  | **Geographical quotas** |
| --- | --- |
| **Germany** | - ≥15% South: Baden-Württemberg, Bayern - ≥25% West: Nordrhein-Westfalen, Hessen, Rheinland-Pfalz, Saarland - ≥10% East: Berlin, Brandenburg, Sachsen, Sachsen-Anhalt, Thüringen - ≥10% North: Schleswig-Holstein, Hamburg, Niedersachsen, Bremen, Mecklenburg-Vorpommern |
| **Italy** | - ≥20% Northwest: Piemonte, Valle d’Aosta, Liguria, Lombardia - ≥10% Northeast: Trentino-Alto Adige/Südtirol, Veneto, Friuli-Venezia Giulia, Emilia-Romagna - ≥10% Central: Toscana, Umbria, Marche, Lazio - ≥15% South: Abruzzo, Molise, Campania, Puglia, Basilicata, Calabria - ≥5% Islands: Sicilia, Sardegna |

Physician specialization quota

| Specialization | Germany | Italy |
| --- | --- | --- |
| Cardiologist | 20% | – |
| Diabetologist | 10% | – |
| General practitioner | 50% | 30% |
| Infectious disease specialist | – | 20% |
| Pulmonologist | 20% | 30% |
| Specialist in hygiene and public health | – | 20% |

This study recruited general practitioners and physicians in specializations that commonly administer or recommend respiratory infection vaccines in the respective countries.

References

1. European Commission. Statistical regions in the European Union and partner countries. NUTS and statistical regions 2021. Luxembourg: Publications Office of the European Union; 2022.
2. COVID-19 Impfquoten-Monitoring in Deutschland (COVIMO). Robert Kock Institut. Report. 2021.
