# Supplementary material for: Physician knowledge, attitudes, and perceptions of respiratory syncytial virus in older adults: A cross-sectional survey in Germany and Italy
Source: PLoS One. 2025 Aug 28;20(8):e0330763. doi: 10.1371/journal.pone.0330763 (PMC12393788; doi:10.1371/journal.pone.0330763)
Supplement: S1 File — S1 Appendix. Sample physician survey in English. Local language versions (German or Italian) were used for data collection. S2 Appendix. Physician quotas targeted for the main survey phase. S1 Fig. Physician sample disposition. S1 Table. Effect of physicians’ characteristics on their knowledge of respiratory vaccination recommendations. S2 Table. Effect of physicians’ characteristics on their knowledge of RSV disease. S3 Table. Physician information needs of respiratory infections, by reported specialization. S4 Table. Physician perceived importance of RSV burden in different patient populations and adults without the listed comorbidities, by country. S5 Table. Effect of physician characteristics on perceived barriers to RSV vaccination. (ZIP) [file pone.0330763.s001.zip › Supporting_Information/S3_Table.docx]

Supplementary Table 3. Physician information needs on respiratory infections, by reported specialisation

| ****Respiratory infection, n (%)**** | Overall  (N=262)^†^ | General practitioner  (n=120) | Pulmonology  (n=58) | ****Cardiology****  **(n=25)** | ****Diabetology****  **(n=10)** | ****Hygiene and public health****  **(n=26)** | ****Infectious disease****  **(n=23)** |
| --- | --- | --- | --- | --- | --- | --- | --- |
| Influenza | 27 (10) | 5 (4.2) | 4 (6.9) | 2 (8.0) | 0 | 12 (46) | 4 (17) |
| COVID-19 | 35 (13) | 9 (7.5) | 9 (16) | 4 (16) | 0 | 12 (46) | 1 (4.3) |
| Pertussis | 73 (28) | 27 (23) | 17 (29) | 7 (28) | 2 (20) | 14 (54) | 6 (26) |
| Pneumococcal disease | 45 (17) | 17 (14) | 8 (14) | 3 (12) | 1 (10) | 12 (46) | 4 (17) |
| RSV | 184 (70) | 91 (76) | 37 (64) | 19 (76) | 7 (70) | 17 (65) | 13 (57) |
| Did not need more information on any of the above | 60 (23) | 26 (22) | 14 (24) | 5 (20) | 3 (30) | 4 (15) | 8 (35) |

^†^Physicians who reported a single specialisation. RSV: respiratory syncytial virus.
