# Supplementary material for: Physician knowledge, attitudes, and perceptions of respiratory syncytial virus in older adults: A cross-sectional survey in Germany and Italy
Source: PLoS One. 2025 Aug 28;20(8):e0330763. doi: 10.1371/journal.pone.0330763 (PMC12393788; doi:10.1371/journal.pone.0330763)
Supplement: S1 File — S1 Appendix. Sample physician survey in English. Local language versions (German or Italian) were used for data collection. S2 Appendix. Physician quotas targeted for the main survey phase. S1 Fig. Physician sample disposition. S1 Table. Effect of physicians’ characteristics on their knowledge of respiratory vaccination recommendations. S2 Table. Effect of physicians’ characteristics on their knowledge of RSV disease. S3 Table. Physician information needs of respiratory infections, by reported specialization. S4 Table. Physician perceived importance of RSV burden in different patient populations and adults without the listed comorbidities, by country. S5 Table. Effect of physician characteristics on perceived barriers to RSV vaccination. (ZIP) [file pone.0330763.s001.zip › Supporting_Information/S4_Table.docx]

Supplementary Table 4. Physician perceived importance of RSV burden in different patient populations and adults without the listed comorbidities, by country

|  | **Germany (n=152)** | **Italy (n=155)** | **p value^†^** |
| --- | --- | --- | --- |
| **Patients with lung disease, n (%)** | | | |
| 50–59 YOA with asthma  Not at all important  Not very important  Somewhat important  Very important  I don’t know | 0  4 (2.6)  39 (26)  107 (70)  2 (1.3) | 0  13 (8.4)  71 (46)  70 (45)  1 (0.6) | <0.001 |
| 50–59 YOA with COPD  Not at all important  Not very important  Somewhat important  Very important  I don’t know | 0  1 (0.7)  31 (20)  117 (77)  3 (2.0) | 0  13 (8.4)  63 (41)  77 (50)  2 (1.3) | <0.001 |
| ≥60 YOA with asthma  Not at all important  Not very important  Somewhat important  Very important  I don’t know | 0 (0)  2 (1.3)  29 (19)  119 (78)  2 (1.3) | 0 (0)  6 (3.9)  62 (40)  86 (55)  1 (0.6) | <0.001 |
| ≥60 YOA with COPD  Not at all important  Not very important  Somewhat important  Very important  I don’t know | 0 (0)  0 (0)  18 (12)  131 (86)  3 (2.0) | 0 (0)  9 (5.8)  39 (25)  106 (68)  1 (0.6) | <0.001 |
| **Patients with cardiovascular disease, n (%)** | | | |
| ≥50 YOA with hypertension  Not at all important  Not very important  Somewhat important  Very important  I don’t know | 4 (2.6)  36 (24)  77 (51)  33 (22)  2 (1.3) | 15 (9.7)  39 (25)  69 (45)  30 (19)  2 (1.3) | 0.114 |
| ≥50 YOA with heart failure  Not at all important  Not very important  Somewhat important  Very important  I don’t know | 1 (0.7)  12 (7.9)  49 (32)  88 (58)  2 (1.3) | 1 (0.6)  14 (9.0)  61 (39)  78 (50)  1 (0.6) | 0.661 |
| ≥50 YOA with coronary heart disease  Not at all important  Not very important  Somewhat important  Very important  I don’t know | 1 (0.7)  18 (12)  59 (39)  72 (47)  2 (1.3) | 1 (0.6)  23 (15)  64 (41)  64 (41)  3 (1.9) | 0.856 |
| **Immunocompromised patients, n (%)** | | | |
| ≥50 YOA with cancer  Not at all important  Not very important  Somewhat important  Very important  I don’t know | 0 (0)  7 (4.6)  42 (28)  101 (66)  2 (1.3) | 0 (0)  17 (11)  51 (33)  86 (55)  1 (0.6) | 0.070 |
| ≥50 YOA who have received an organ transplant and currently receiving immunosuppressant therapy  Not at all important  Not very important  Somewhat important  Very important  I don’t know | 0 (0)  0 (0)  9 (5.9)  140 (92)  3 (2.0) | 0 (0)  5 (3.2)  25 (16)  122 (79)  3 (1.9) | 0.001 |
| ≥50 YOA with HIV infection  Not at all important  Not very important  Somewhat important  Very important  I don’t know | 0 (0)  0 (0)  10 (6.6)  138 (91)  4 (2.6) | 0 (0)  13 (8.4)  34 (22)  105 (68)  3 (1.9) | <0.001 |
| ≥50 YOA treated with any immunosuppressant drug for  immune-related disease  Not at all important  Not very important  Somewhat important  Very important  I don’t know | 0 (0)  0 (0)  9 (5.9)  140 (92)  3 (2.0) | 0 (0)  4 (2.6)  48 (31)  102 (66)  1 (0.6) | <0.001 |
| **Adults without the listed comorbidities, n (%)** | | | |
| 50–59 YOA  Not at all important  Not very important  Somewhat important  Very important  I don’t know | 17 (11)  55 (36)  61 (40)  17 (11)  2 (1.3) | 38 (25)  54 (35)  46 (30)  16 (10)  1 (0.6) | 0.024 |
| 60–64 YOA  Not at all important  Not very important  Somewhat important  Very important  I don’t know | 10 (6.6)  36 (24)  71 (47)  33 (22)  2 (1.3) | 21 (14)  46 (30)  67 (43)  20 (13)  1 (0.6) | 0.056 |
| 65–69 YOA  Not at all important  Not very important  Somewhat important  Very important  I don’t know | 8 (5.3)  22 (14)  67 (44)  53 (35)  2 (1.3) | 10 (6.5)  34 (22)  71 (46)  38 (25)  2 (1.3) | 0.238 |
| ≥70 YOA  Not at all important  Not very important  Somewhat important  Very important  I don’t know | 4 (2.6)  15 (9.9)  42 (28)  88 (58)  3 (2.0) | 3 (1.9)  21 (14)  57 (37)  72 (46)  2 (1.3) | 0.260 |

^†^p values determined with Fisher’s exact test. COPD: chronic obstructive pulmonary disease; HIV: human immunodeficiency virus; RSV: respiratory syncytial virus; YOA: years of age.
